# Differential roles of 3-Hydroxyflavone and 7-Hydroxyflavone against nicotine-induced oxidative stress in rat renal proximal tubule cells

**DOI:** 10.1371/journal.pone.0179777

**Published:** 2017-06-22

**Authors:** Bidisha Sengupta, Mehdi Sahihi, Monireh Dehkhodaei, Darrian Kelly, Istvan Arany

**Affiliations:** 1 Department of Chemistry, Tougaloo College, Tougaloo, Mississippi, United States of America; 2 Department of Chemistry, University of Isfahan, Isfahan, Iran; 3 Department of Pediatrics, University of Mississippi Medical Center, Jackson, Mississippi, United States of America; Jadavpur University, INDIA

## Abstract

Plant flavonoids are well known as antioxidants against oxidative stress induced by exposure to external pollutants. Nicotine (NIC) is one of those agents which increases renal oxidative stress, an important factor in the pathogenesis of renal epithelial injury in smokers. Although several studies had been conducted on flavonoids and oxidative stress, the mechanism of the protective pathways are not fully understood. Here, we present studies on antioxidant properties of two mono-hydroxyflavone isomers, 3-hydroxyflanove (3HF)- and 7-hydroxyflavone (7HF), against nicotine-associated oxidative stress and injury in cultured renal proximal tubule cells and correlate their antioxidant properties with their chemical structure. Our data clearly demonstrates, for the first time, that while both 3HF and 7HF protect renal cells from NIC-associated cytotoxicity, the mechanism of their action is different: 3HF elicits protective activity via the PKA/CREB/MnSOD pathway while 7HF does so via the ERK/Nrf2/HO-1 pathway. Molecular docking and dynamics simulations with two major signaling pathway proteins showed significant differences in the binding energies of 3HF (-5.67 and -7.39 kcal.mol^-1^) compared to 7HF (-5.41 and -8.55 kcal.mol^-1^) in the matrices of CREB and Keap1-Nrf2 proteins respectively, which corroborate with the observed differences in their protective properties in the renal cells. The implications of this novel explorative study is likely to promote the understanding of the mechanisms of the antioxidative functions of different flavones.

## Introduction

Nicotine (NIC) is a major tobacco alkaloid and component of E-cigarettes [1]. NIC links smoking to renal injury via increased oxidative stress [2]. Our *in vitro* studies confirmed that NIC stimulates mitochondrial ROS production, which leads to a mitochondrial depolarization-dependent injury of renal proximal tubule cells [3, 4]. Previously we have also shown that NIC exposure increases oxidative stress in the kidneys of mice, leading to sub-lethal injury of the kidney [4]. Since the popularity of E-cigarettes is on the rise, it may further increase renal risk in the relevant population [5, 6]. Therefore, modalities that ameliorate smoking/nicotine exposure-associated renal oxidative stress are of high importance.

Flavonols and related phenolic compounds of the flavonoid group are ubiquitous in plants of higher genera and are abundant in common plant-based foods and beverages such as citrus fruits, apple, strawberry, soy products, onion, broccoli, tea and red wine [7–9]. Flavonoids protect various cell types from oxidative stress via different mechanisms. The most recognized mechanism is their direct antioxidant activity, which involves scavenging of reactive oxygen species (ROS) and peroxynitrite [10]. Additionally, flavonoids elicit indirect antioxidant activity through transcriptional induction of genes with antioxidant properties such as heme oxygenase-1 (HO-1) [10] or the mitochondrial manganese superoxide dismutase (MnSOD) [11].

Both *in vivo* and *in vitro* studies show that flavonoids are therapeutically effective against a wide range of free radical mediated disorders including atherosclerosis, ischemia, neuronal degeneration, and cancer [8, 9, 11–14]. Some studies raised the possibility that direct (scavenging) and/or indirect (induction of antioxidant enzymes) antioxidant activities of flavonoids are determined by the extent and nature of their hydroxylation [15].

**Fig 1:**
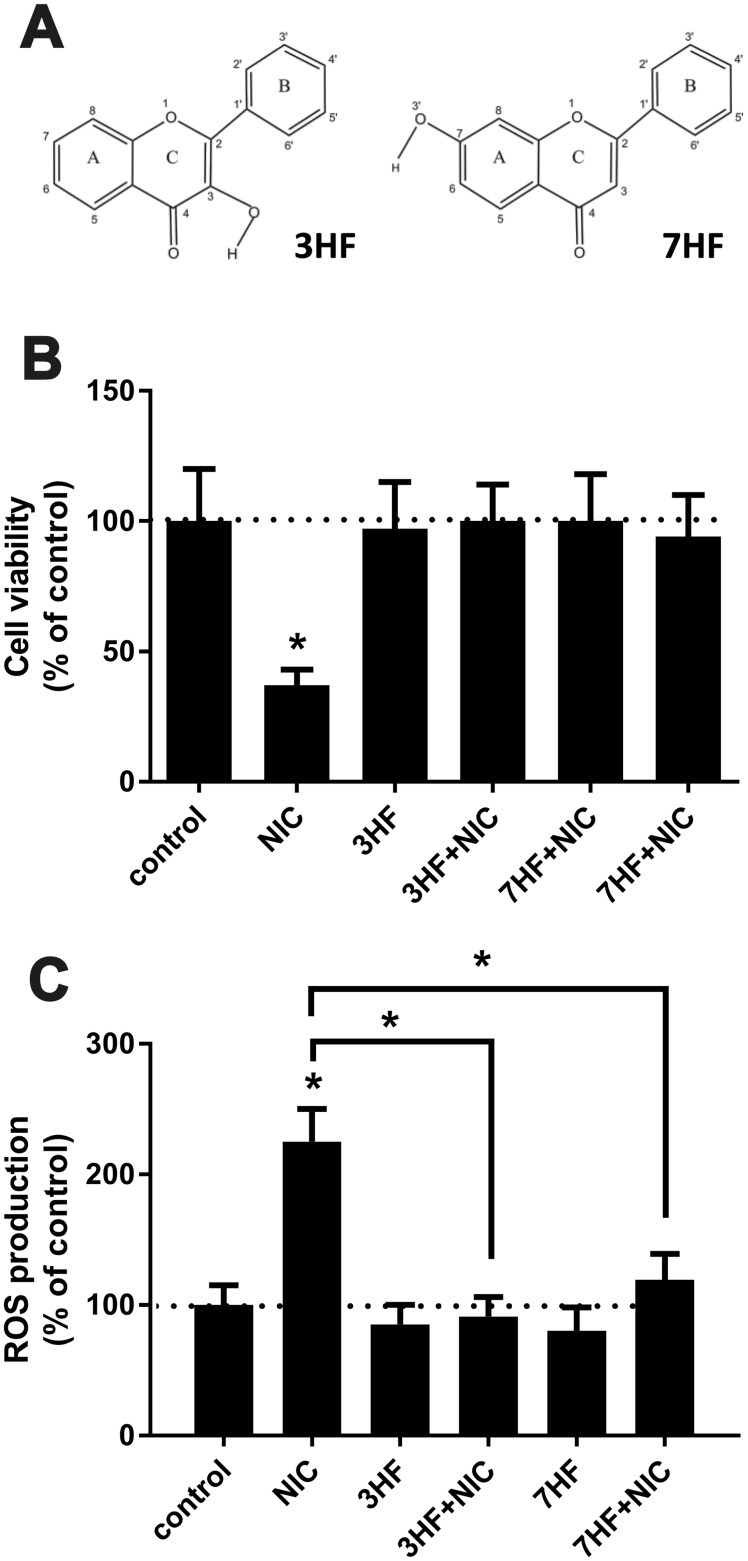
3HF and 7HF rescues renal proximal tubule cells from nicotine exposure-associated cytotoxicity by inhibiting ROS production. (A) Chemical structure of 3-hydroxyflavone (3HF) and 7-hydroxyflavone (7HF). (B) NRK52E cells were pretreated with 20 μM 3HF or 7HF overnight prior to treatment with 200 μM NIC and cell viability was determined 24 hours later. Control cells were left untreated, or treated with 200 μM NIC, 20 μM 3HF or 7HF. Values were expressed as % of untreated control. n = 3; *p<0.05 compared to untreated control. Dotted line represents untreated control value. (C) NRK52E cells were pretreated with 20 μM 3HF or 7HF overnight and 200 μM NIC-mediated ROS production was determined. Control cells were left untreated, or treated with 200 μM NIC, 20 μM 3HF or 7HF. Values are expressed as % of untreated control. n = 3; *p<0.05 compared to untreated control or as indicated. Dotted line represents untreated control value.

Flavonoids are comprised of a common structure of diphenylpropane (Fig 1A) C6-C3-C6, consisting of two aromatic rings (rings A and B) linked through a three carbon bridge or by a pyrone or pyrane ring (ring C) [16]. Most of the existing studies in the literature provide evidence of the potential benefits of the -OH groups in the 'B'-ring [8, 9, 12, 13]. However, the influence of only the 'A'/'C'-ring-OH group(s) is still unknown.

The present study determines for the first time, the differential roles of the hydroxyl group at the 'A' and 'C'-rings of 7-hydroxyflavone (7HF) and 3-hydroxyflavone (3HF) respectively, as antioxidants (structures shown in Fig 1A). 7HF and 3HF are the synthetic chromophores and prototypes of all naturally occurring flavonoids. Here, we determined the indirect antioxidant [17] function of 3HF and 7HF using rat renal proximal tubule (NRK52E) cells treated with nicotine. Molecular docking and dynamics simulations determined the differential interactions, stabilities and dynamics of the flavones 3HF and 7HF with two major signaling proteins, cAMP responsive element-binding protein (CREB) [18] and Nrf2-Keap1 (nuclear factor erythroid 2 [NF-E2]-related factor 2 [Nrf2])–Keap1 (Kelch-like erythroid cell-derived protein with CNC homology [ECH]-associated protein 1) [19] and correlated with their antioxidant properties.

## Materials and methods

### Cell line and treatment

The rat renal proximal tubule cell line (NRK52E) was purchased from ATCC (Manassas, VA) and maintained as suggested. 3-hydroxyflavone (3HF) and 7-hydroxyflavone (7HF) were obtained from Sigma-Aldrich, St. Louis, MO, and were applied in 20 μM concentration overnight prior to treatment with 200 μM nicotine (Sigma-Aldrich, St. Louis, MO). The dose of NIC was chosen based on our earlier studies that showed that this dose effectively increases reactive oxygen species (ROS) production in cultured renal proximal tubule cells [4]. The most effective concentration of flavones was found to be ~ 20 μM (data not shown), which agrees with the published IC50 values [20] and concentration used in similar studies [21].

### Viability assay

To assess cellular viability, the “Cell Titer Blue” fluorescent kit was used (Promega, Madison, WI). This assay is based on the ability of living cells to convert a redox dye (resazurin) into a fluorescent end product (resorufin). Nonviable cells rapidly lose metabolic capacity and thus do not generate a fluorescent signal. Briefly: cells grown in 96-well-plates were treated as needed and the redox dye (resazurin) was added. After 2 hours of incubation fluorescence was measured using a fluorescent plate-reader (Fluorocount, Packard, Cole-Palmer, Vernon Hills, IL). Data expressed as arbitrary units of fluorescence or as percentage of fluorescence in the control cells.

### Measurement of intracellular ROS production

Microplate assay using oxidant-sensitive 2′,7′-dichlorofluorescein-diacetate (DCFDA; Invitrogen, Grand Island, NY) measured the intracellular generation of ROS. Cells grown in T25 flasks were pretreated with either 20 μM 3HF or 20 μM 7HF for overnight as required and isolated with trypsinization. After washing and counting, cells were loaded with 100 μM DCFDA in HBSS for 30 min at 37°C. After incubation, the excess dye was removed by washing with fresh HBSS and placed in wells of a 96-well plate (0.5 × 10^6^ cells/well). 200 μM NIC was added to the appropriate wells and the increase in fluorescence was monitored in a fluorescence plate reader (Fluorocount, Packard) at 485 nm_exc_/530 nm_em_. ROS production was calculated as changes in fluorescence/30 min/0.5 × 10^6^ cells and expressed as the percentage of untreated values as described in our earlier studies [22].

### Manipulation of Nrf2 or MnSOD expression as well as ERK or CREB activation

Nrf2 expression was knocked down by a Nrf2 siRNA (Santa Cruz, Ja Jolla, CA) using Lipofectamine 3000 (Life Technologies, Grand Island, NY) as we reported earlier [23]. To knockdown MnSOD expression, NRK52E cells were transfected with a short-hairpin (sh)MnSOD plasmid (Addgene, Cambridge, MA, USA) using Lipofectamine 3000 as we described elsewhere [24]. ERK and CREB activations were inhibited by infection with a dominant negative MEK1 (dnMEK) or M1CREB adenovirus, respectively, as described in our previous studies [25, 26].

### Reporter luciferase assay

NRK52E cells grown in 24-well-plates were transfected with either of the following reporter luciferase plasmids: MnSOD-promoter-reporter-luciferase [27], HO-1 promoter luciferase [28], CRE luciferase (Stratagene/Agilent Technologies, Santa Clara, CA) and ARE luciferase (Qiagen, Germantown, MD) together with a Renilla luciferase (Promega, Madison, WI) by using Lipofectamine 3000 reagent (Life Technologies, Grand Island, NY). 24 hours after treatment(s) firefly and renilla luciferase activities were determined by using the Dual Luciferase assay kit (Promega, Madison, WI, cat# E2920). Luciferase activities were calculated as ratios of the firefly and renilla activities and expressed as percentage of the control (untreated) values.

### Preparation of the proteins and ligands for computational studies

Structures of 3HF and 7HF were drawn using ChemDraw 12 and Becke three-parameter Lee–Yang–Parr (B3LYP) hybrid density functional theory with the basis set of 6–31 g** was employed to optimize the flavonols structures, using the GAMESS quantum chemistry software (USA) [29]. The known crystal structures of the proteins Keap1-Nrf2 conjugate (PDB ID: 2DYH) [30] and cAMP responsive element-binding protein (CREB) (PDB ID: 1DH3) [31] were obtained from the RCSB Protein Data Bank at resolution of 1.9 Å and 3.0 Å for 2dyh and 1DH3, respectively. These structures were energetically optimized by applying the gromos96 43a1 force field [32] available in GROMACS package 5.1.2 [33].

### Molecular docking procedure

In this work, docking study was carried out to determine the binding affinities and sites of 3HF and 7HF in Keap1-Nrf2 conjugate and CREB. Water molecules of the protein.pdb files were removed, missing hydrogen atoms and Gasteiger charges were added. Flexible-ligand docking was performed by AutoDock 4.2 molecular-docking program using the implemented empirical free energy function and the Lamarckian Genetic Algorithm [34]. The Auto Grid was used to calculate Grids and a blind docking with 126 lattice points along X, Y, and Z axes was performed to find the active site of ligands to the protein. After determination of the active site, the dimensions of the grid map were selected 60 points with a grid point spacing of 0.375 Å, to allow the ligand to rotate freely. 250 docking runs with 25,000,000 energy evaluations for each run were performed.

### Molecular dynamics (MD) simulation

MD simulation [35–37] method was used to compare the structural changes of proteins in the absence and presence of 3HF and 7HF. The flavonol-protein complexes with the most negative free binding energy were considered as the initial conformations for the MD studies. GROMACS 5.1.2 package and gromos96 43a1 force field were used to carry out all MD studies. Free proteins and flavonol-protein complexes were located in the cubic box with the periodic boundary conditions in the three directions. The solutes were placed in the center of box and the minimum distance between solute surface and the box was 1.0 nm. The box filled with SPC water molecules [38, 39], and the solvated systems were neutralized by adding appropriate amounts of sodium ions (Na^+^) and chloride ions (Cl^-^). After energy minimization using the steepest descent method, the systems were equilibrated for 200 ps at the temperature of 300K. Finally, an 18 ns MD simulation was carried out at 1bar and 300K. Parrinello-Rahman barostate [40] at 1 bar, Berendsen thermostat [41] at 300K, 9 Å cut off for van der Walls and Coulomb interactions and the particle mesh Ewald (PME) method [42, 43] for long range electrostatics were used. The leap-frog algorithm with the 2 fs time step was used to integrate the equation of motions. Finally, an all-bond constrain was used to keep the ligand from drifting and the atomic coordinates were recorded to the trajectory file every 0.5 ps for later analysis.

## Results

### 3HF or 7HF prevents nicotine-associated cell injury by suppressing nicotine-induced ROS production in renal proximal tubule cells

To determine the beneficial effects of 3HF and 7HF on NIC-induced cell injury, renal proximal tubule (NRK52E) cells were pre-treated with 20 μM 3HF or 7HF overnight prior to treatment with 200 μM NIC and cell viability was determined 24 hours later. Fig 1B shows that NIC significantly decreases cell viability, which was prevented by pretreatment with either 3HF or 7HF (Fig 1B). We also showed that neither 3HF nor 7HF elicits any cytotoxicity at this concentration (Fig 1B). Since cytotoxic effects of NIC are associated with its capability to produce excessive amounts of ROS in renal proximal tubule cells [3, 4], we tested whether these hydroxyflavones affect NIC-mediated ROS production? Accordingly, NRK52E cells were pretreated with either 20 μM 3HF or 7HF overnight and 200 μM NIC-mediated ROS production was determined as described in Materials and Methods. As is shown in Fig 1C, both 3HF and 7HF significantly attenuated NIC-induced ROS production. In fact, 3HF or 7HF treatment alone exerts some antioxidant effects via reducing basal ROS production (Fig 1C). It is important to note that the ROS-inhibiting effect of 3HF is moderately but significantly stronger than 7HF at an equimolar concentration.

#### 3HF and 7HF activates the promoter of distinct antioxidant genes

**Fig 2:**
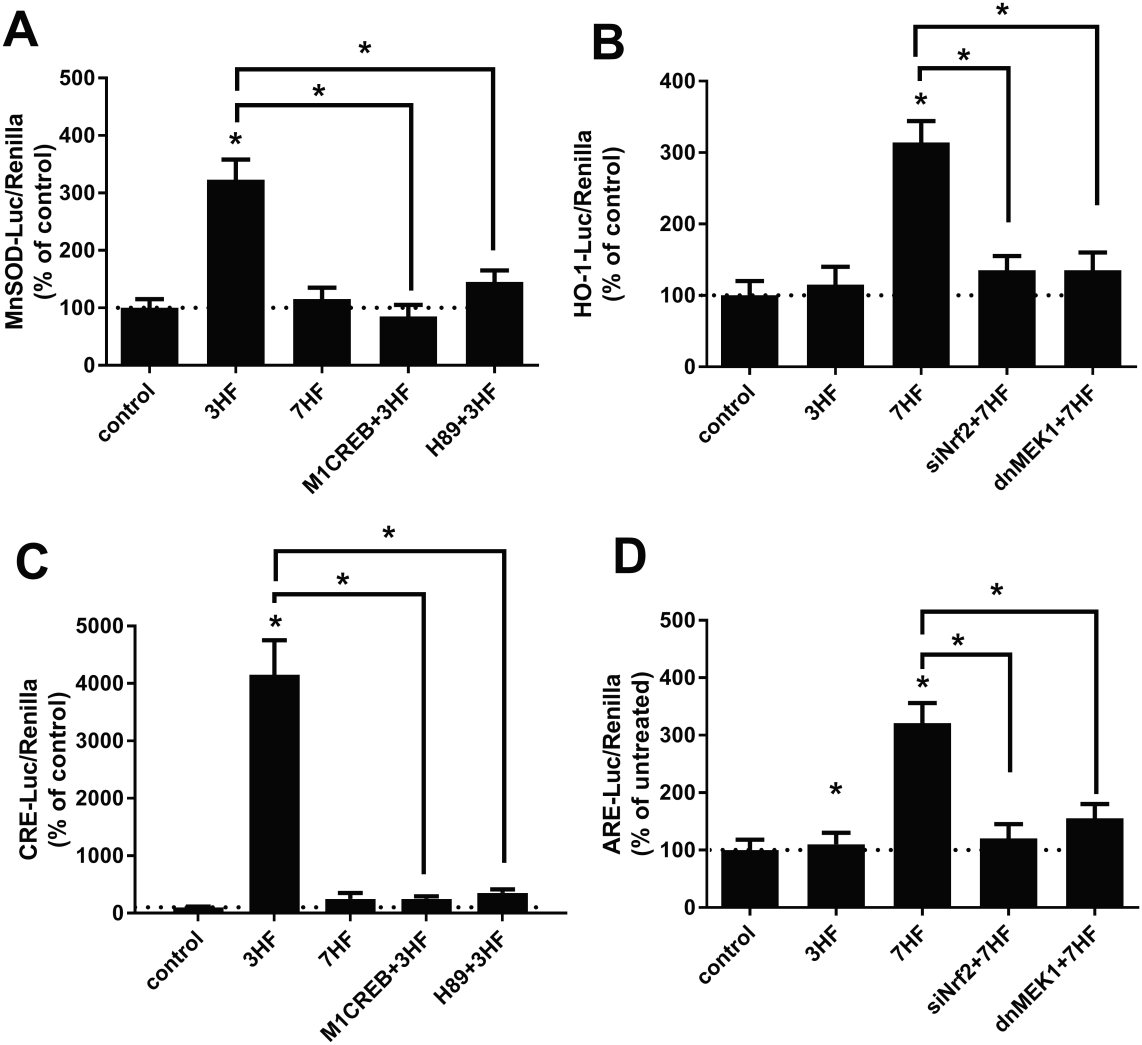
3HF and 7HF activates distinct antioxidant genes via distinct signaling pathways. (A) NRK52E cells were transfected with a MnSOD promoter luciferase plasmid as described in Materials and Methods. A set of cells were infected with the M1CREB adenovirus overnight or treated with 10 μM H89 1 hr prior to treatment with 20 μM 3HF or 7HF. Luciferase activities were determined 24 hours later. n = 3; *p<0.05 compared to control or as indicated. Dotted line represents control value. (B) NRK52E cells were transfected with an HO-1 promoter luciferase plasmid as described in Materials and Methods. A set of cells were co-transfected with 20 nM Nrf2 siRNA or infected with the dnMEK adenovirus 24 hours prior to treatment with 20 μM 3HF or 7HF. Luciferase activities were determined 24 hours later. n = 3; *p<0.05 compared to control or as indicated. Dotted line represents control value. (C) NRK52E cells were transfected with a CRE luciferase plasmid as described in Materials and Methods. A set of cells were infected with a M1CREB adenovirus 24 hours or treated with 10 μM H89 1 hr prior to treatment with 20 μM 3HF or 7HF. Luciferase activities were determined 24 hours later. n = 3; *p<0.05 compared to control or as indicated. Dotted line represents control value. (D) NRK52E cells were transfected with an ARE luciferase plasmid as described in Materials and Methods. A set of cells were co-transfected with 20 nM Nrf2 siRNA or infected with the dnMEK adenovirus 24 hours prior to treatment with 20 μM 3HF or 7HF. Luciferase activities were determined 24 hours later. n = 3; *p<0.05 compared to control or as indicated. Dotted line represents control value.

A major mechanism by which flavonoids exert their indirect antioxidant properties is the induction of antioxidant genes such as HO-1 and MnSOD [44]. To test this, NRK52E cells were transfected with either a MnSOD or a HO-1 promoter luciferase plasmid together with a renilla luciferase and treated with 20 μM 3HF or 7HF. 24 hours later luciferase activities were determined as described in Materials and Methods. Interestingly, 3HF preferably activates the MnSOD promoter (Fig 2A) while 7HF, rather the HO-1 promoter (Fig 2B). Since both MnSOD and HO-1 can be activated via the antioxidant response element (ARE) [44], activation of an ARE reporter luciferase was also determined after treatment with 3HF or 7HF. As shown in Fig 2D, ARE was preferably induced by 7HF. Instead of the ARE 3HF preferably induced a CRE reporter luciferase (Fig 2C). The CRE element binds the transcription factor CREB and induces transcription of the MnSOD [45]. Indeed, infection of NRK52E cells with a dominant-negative CREB (M1CREB) adenovirus inhibited 3HF-mediated induction of the MnSOD promoter (Fig 2A) and a CRE reporter (Fig 2C). In addition, the protein kinase A (PKA) inhibitor H89 also significantly attenuated 3HF-mediated induction of the MnSOD promoter (Fig 2A) and the CRE reporter (Fig 2C). In contrast, knock-down of endogenous Nrf2 (a transcription factor that binds the ARE [44]) via a Nrf2 siRNA (siNrf2) did not elicit significant effects (data not shown). These data suggest that 3HF induces the MnSOD promoter through the PKA/CREB/CRE axis and not through Nrf2/ARE.

We also determined whether activation of Nrf2/ARE (Fig 2D) is responsible for 7HF-mediated induction of the HO-1 promoter. Accordingly, NRK52E cells were transfected with a Nrf2 siRNA (siNrf2) together with either an HO-1 promoter or ARE reporter and treated with 20 μM 7HF. 24 hours later luciferase activities were determined. Fig 2B shows that knockdown of Nrf2 significantly attenuated activity of the HO-1 promoter (Fig 2B) and the ARE reporter (Fig 2D). In addition, inhibition of ERK via a dominant-negative MEK (dnMEK) adenovirus, also inhibited 7HF-mediated activation of the HO-1 promoter (Fig 2B) and the ARE reporter (Fig 2D). In contrast, neither M1CREB nor H89 did not affect 7HF-mediated activation of HO-1 and the ARE (data not shown). These results suggest that 7HF activates the HO-1 promoter via the ERK/Nrf2/ARE axis.

#### Protective effects of 3HF and 7HF requires activation of MnSOD or HO-1, respectively

**Fig 3:**
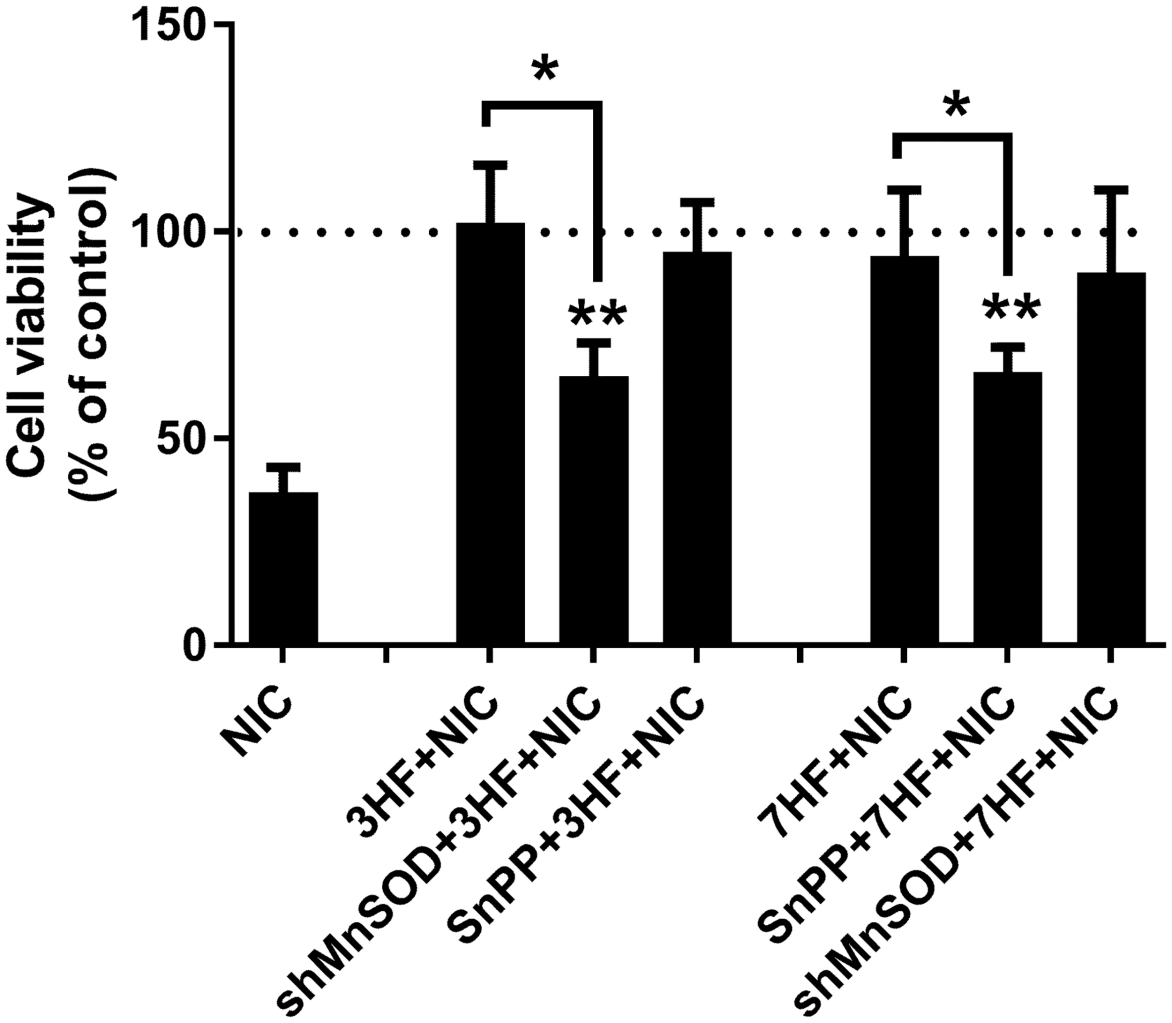
3HF and 7HF rescues renal proximal tubule cells from nicotine-induced cytotoxicity via induction of MnSOD or HO-1, respectively. NRK52E cells were transfected with an shMnSOD plasmid 24 hours or pre-treated with 10 μM SnPP 1 hr prior-to treatment with 20 μM 3HF or 7HF overnight followed by treatment with 200 μM NIC. 24 hours later cell viablity was determined. n = 3, *p<0.05 as indicated; **p<0.05 compared to NIC-treated value.

To demonstrate that 3HF and 7HF rescues renal proximal tubule cells from NIC-dependent cytotoxicity via activation of the anti-oxidant MnSOD or HO-1, respectively, the following experiments were performed. NRK52E cells were transfected with a short-hairpin MnSOD (shMnSOD) to knockdown MnSOD expression or treated with 10 μM tin-protoporphyrine (SnPP) to inhibit HO-1 activity then treated with either 20 μM 3HF or 20 μM 7HF overnight followed by 200 μM NIC. 24 hours later cell viability was determined. Fig 3 shows that knockdown of MnSOD (shMnSOD) significantly attenuated beneficial effects of 3HF but not 7HF. In contrast, inhibition of HO-1 activity (SnPP) significantly attenuated beneficial effects of 7HF but not 3HF. These data suggest that protective effects of 3HF is due to—at least partly- activation of MnSOD while protective effects of 7HF due to—at least partly- activation of HO-1.

### Computational studies

Cellular studies suggested the protective roles of 3HF and 7HF against NIC-induced oxidative stress through the induction of antioxidant enzyme through different mechanisms that involves Nrf2 and CREB. Hence, it is pertinent [21] to study the binding interactions and molecular dynamics of 3HF and 7HF with transcription related proteins such as Keap1-Nrf2 conjugate (pdb id: 2DYH) and CREB (pdb id: 1DH3).

### Molecular docking studies using AutoDock

**Fig 4:**
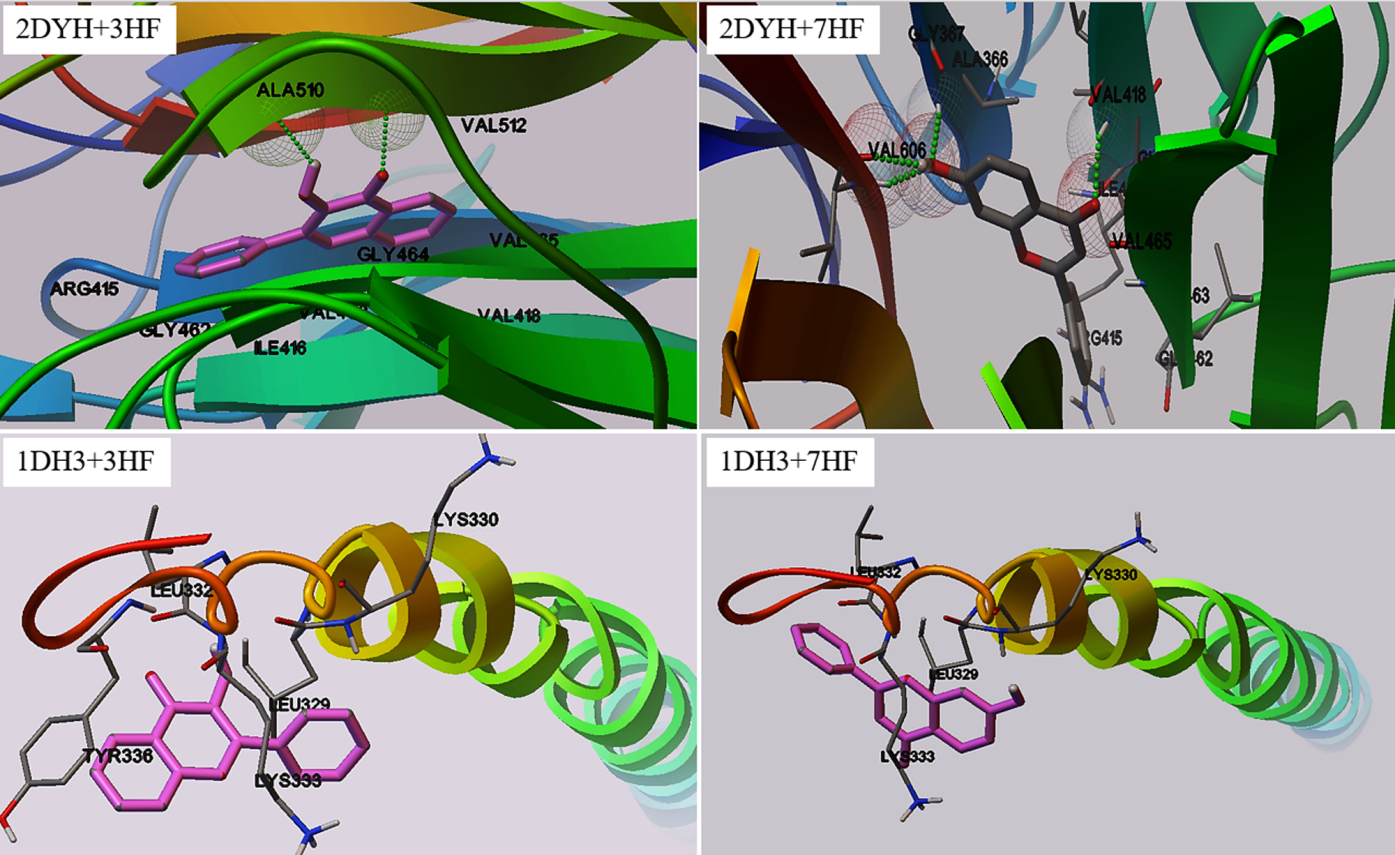
Binding sites. The docking sites of 3HF and 7HF on 2DYH and 1DH3 proteins.

Crystal structure analysis revealed that 2DYH consist of two polypeptide chains [30], while 1DH3 exist as a monomer or a dimer conjugated with DNA [31]. We chose the monomer of 1DH3 for the present study. AutoDock program was chosen to examine the binding modes of 3HF and 7HF with the transcription factors. The best score ranked results are shown in Table 1. The docking results showed that although there is not a major difference in the types of binding interactions, 3HF binds to 1DH3 with a 4.8% more binding energy than 7HF. On the contrary, the binding energy of 7HF is 15.7% more than 3HF in the case of 2DYH, with different binding modes. Fig 4 shows the binding sites of the flavones in the protein matrices. 7HF is in H-bond interaction with Val(418), Val(606) and Gly(367) of 2DYH. Also, H-bond interactions with Ala(510) and Val(512) stabilized 3HF in the binding site of 2DYH. These results clearly indicate that the -OH in the 'A' ring act differently than the 'C' ring in flavone toward interacting with the signaling proteins, which warrant a detailed study using molecular dynamics simulations.

**Table 1:** Docking summary of 7HF and 3HF with 2DYH and 1DH3 proteins.

System	Binding energy (kcal.mol^-1)^	Amino acids around the ligand	Type of interaction		
			H- bond	π -π	π -Cation
2DYH-7HF	-8.55	GLY367- ALA366- VAL606-VAL418- GLY417- ILE416- VAL465-VAL463- ALA510- GLY462- ARG415- GLY509	4	NO	NO
2DYH-3HF	-7.39	ALA366- ILE416- ARG415- VAL418- VAL463- GLY462- ALA510- GLY464- VAL465- VAL512	2	NO	NO
1DH3-Monomer 7HF	-5.41	LYS330- LYS333- LEU332- LEU329	NO	NO	NO
1DH3-Monomer 3HF	-5.67	TYR336- LYS330- LYS333- LEU332- LEU329	NO	NO	NO

### Molecular dynamics simulation studies

**Fig 5:**
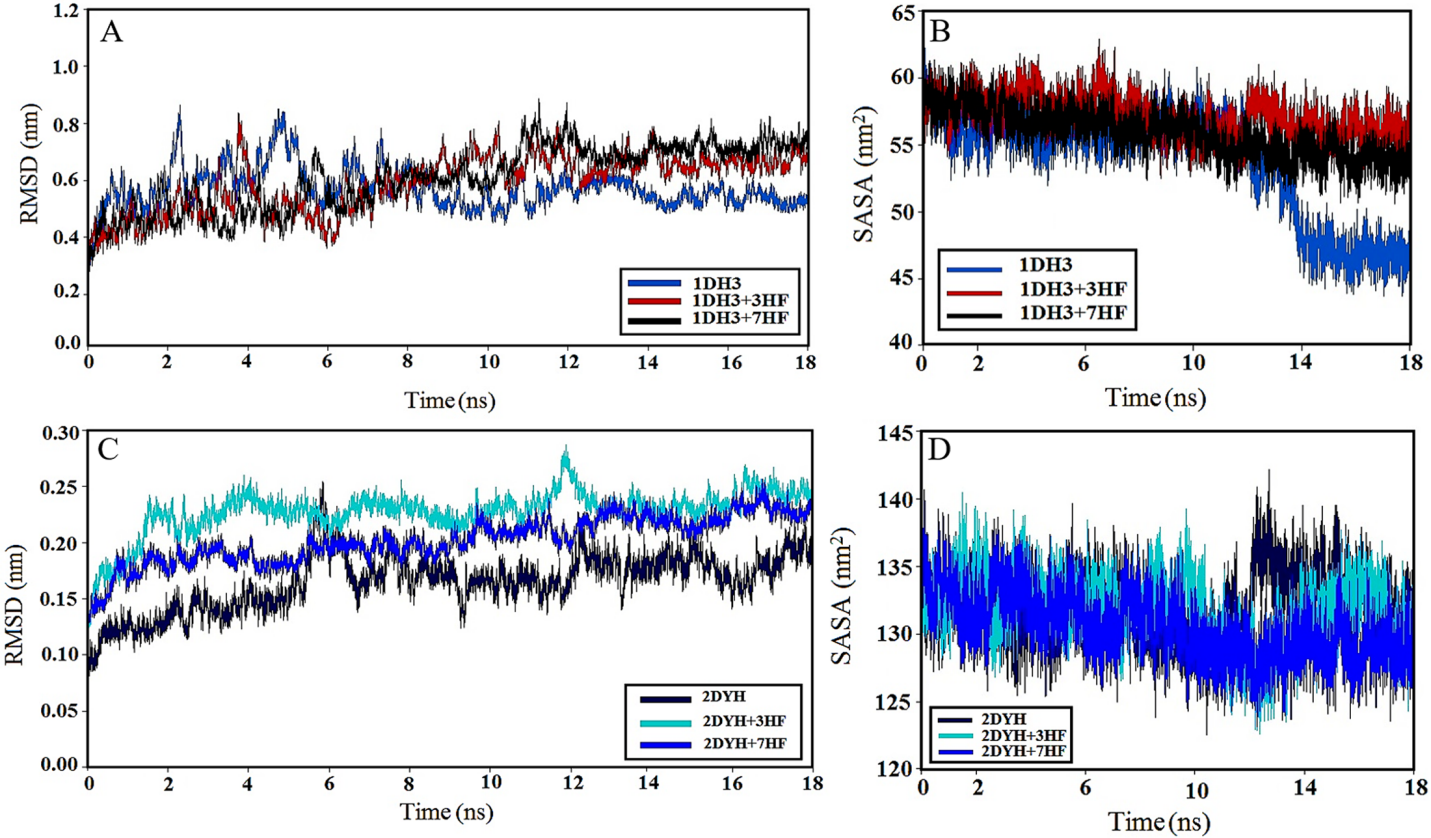
Molecular dynamics simulations. (A) Time dependence of RMS deviation of distance (RMSD) between alpha-carbon atoms from the crystal structure as a function of simulation time for free 1DH3 and conjugated 1DH3. (B) Time evolution of the solvent accessible surface area (SASA) during 18 ns of MD simulation of 1DH3, 1DH3 with 3HF and 1DH3 with 7HF. (C) Time dependence of RMS deviation of distance (RMSD) between alpha-carbon atoms from the crystal structure as a function of simulation time for free 2DYH and conjugated 2DYH. (D) Time evolution of the solvent accessible surface area (SASA) during 18 ns of MD simulation of 2DYH, 2DYH with 3HF and 2DYH with 7HF. Color codes are given on the figures.

The beginning structures for the MD analyses were selected from the conformations with lowest docking energies. The stability of the system (protein and ligand) properties was examined by means of RMS deviations (RMSD) of unbound and ligand conjugated protein with respect to the initial structure, RMS fluctuations (RMSF), the solvent accessible surface area (SASA) of protein and number of H-bonds. Preliminary simulations over 18 ns time were performed on 1DH3 and 2DYH with ligands 3HF and 7HF. Table 2 provides the average values of RMSD, SASA and number of H-bonds of the systems and Fig 5 presents time dependence of RMSD and SASA values of 1DH3, 1DH3-ligands, 2DYH and 2DYH-ligands systems. Fig 5A and 5C indicate that the trajectories of all systems are stable and their RMSD reached equilibrium and fluctuated around its mean value after about 5 ns simulation time. The average RMSD of 3HF-1DH3 and 7HF-2DYH are closer to free protein than the other conjugates (Table 2). Fig 5B shows that complexation with 3HF improved the SASA of 1DH3 when compared to free and 7HF bound protein, which might be associated to its functionalities. On the contrary, not a significant change in SASA of 2DYH was observed in the presence of ligands (Fig 5D and Table 2). Local protein mobility was analyzed by calculating the time averaged RMSF values of free protein and protein-flavonol complexes and were plotted against residue numbers based on the last 10000 ps trajectory (S1 Fig). The profiles of atomic fluctuations were found to be very similar to those of free protein and protein-flavonol complexes. RMSF highlights the conformational adjustments of the protein structure, in conjunction with ligand conformational adaptation to their binding sites. The results indicate that the residues that were in contact with the ligands are stable and have low RMSF values suggesting that the structure of drug binding site remains rigid during simulation. In addition, the RMSF of the atomic positions of the ligands was calculated to examine their conformational variations (S2 Fig). The results indicate that the ligand atoms showed limited fluctuations. Hence, it can be concluded that the interactions of proteins and the ligands were stable during the simulation time. The changes in the number of intramolecular H-bonds of proteins during the 18 ns simulation time are shown in Table 2. The variations of H-bonds were similar to the variations of SASA. This similarity confirms the correctness of the MD simulation results. All of the above simulations reveal that the presence of the -OH group in 'A' vs 'C' ring in flavone, significantly influence the microenvironment of the transcription related proteins to different extent, thereby exerting variations in their response against oxidative stress.

**Table 2:** Average values of RMSD, SASA and no. of H-bonds values of 1DH3 and 2DYH proteins in free and 3HF / 7HF bound systems.

System	RMSD (nm)	SASA (nm^2^)	No. of H-bond
2DYH	0.176±0.017	1.79±0.01	221±8
2DYH+3HF	0.103±0.027	1.78±0.01	221±9
2DYH+7HF	0.211±0.015	1.78±0.01	225±8
1DH3	0.345±0.039	2.33±0.03	50±4
1DH3+3HF	0.464±0.041	2.20±0.04	46±3
1DH3+7HF	0.522±0.034	2.11±0.03	48±3

## Discussions

Smoking and E-cigarette use—via their NIC content- increases renal oxidative stress in experimental systems [4] and in the renal patient [46]. While the easiest way to circumvent this problem is smoking cessation, many smokers refused to quit. Hence, therapeutic means are necessary to ameliorate smoking/NIC exposure associated renal oxidative stress and consequent injury. Protective role of antioxidants in smoking/NIC exposure-associated renal injury has been established [47]. Flavonoids—that are abundant in various fruits and vegetables- are known antioxidants in a variety of *in vitro* systems [48]. It has been suggested that flavonols positively affect renal health through their antioxidant function [49, 50]. However, there are virtually no data regarding the impact of flavonoids on renal health during smoking. Protective effect of epicatechin, a potent antioxidant flavonoid in the human diet, on NIC-induced renal oxidative stress has been demonstrated in rats [51]. Hence, it is highly plausible that flavonoids are good candidates to ameliorate NIC exposure-associated renal injury.

Flavonoids exert their beneficial effects—among the others- via “direct” and “indirect” antioxidant activity [10, 15]. Direct antioxidant activity involves scavenging of reactive oxygen (ROS) and reactive nitrogen (RNS) species [10], while their indirect antioxidant effect means induction of enzymes that protect cells from deleterious effects of oxidative stress [10]. Such enzymes include HO-1 or MnSOD expression of which is regulated through the ARE in their promoter proximal region [10].

Interestingly, studies described correlation between the number/configuration of hydroxyl groups and direct antioxidant (scavenging) activity of various flavonoids [15, 52]. However, the relationship between *indirect antioxidant activity* and *configuration* of hydroxyl groups is virtually unknown. In this study we compared indirect antioxidant activity of 3HF and 7HF: both possess only one hydroxyl group but at different position (Fig 1).

Our studies confirmed protective effects of both 3HF and 7HF on NIC-induced cytotoxicity in cultured renal proximal tubule cells (Fig 1B). We also demonstrated that this protection is associated with inhibition of NIC-induced ROS production (Fig 1C). We also proved that protective effects of 3HF is—at least partly- due to induction of MnSOD (Fig 3), while protective effects of 7HF involves induction of the HO-1 gene (Fig 3). It seems that while 3HF and 7HF provides total protection against NIC-mediated cytotoxicity (Fig 1B), protective effects of MnSOD (induced by 3HF) or HO-1 (induced by 7HF) are less effective (Fig 3). It is plausible that protective effects of 3HF and 7HF may be also due to other mechanisms which include inhibition of pro-oxidant genes [10]. This possibility may warrant further investigation.

In addition, we observed that 3HF transcriptionally activates the MnSOD gene via a CRE element (Fig 2C) through inducing the CREB transcription factor by PKA (Fig 2A). In contrast, 7HF activates the promoter of the HO-1 gene via ARE (Fig 2B) through inducing the Nrf2 transcription factor (Fig 2D).

The differential influences of the 3-OH and 7-OH groups in flavones are corroborated by docking and molecular dynamics simulations in the CREB (pdb id: 1DH3) and Nrf2-Keap (pdb id: 2DYH) proteins. The three dimensional structure of the signaling proteins creates preferences for binding to one flavone over another, e.g. 1DH3 prefers 3HF, while 7HF is favored by 2DYH, as is observed through the binding energies in Table 1. Furthermore, molecular dynamics simulations (Table 2 and Fig 5) determined that the root-mean-square deviations of proteins 1DH3 and 2DYH were minimum with ligand 3HF and 7HF respectively, when compared to free protein. Less deviation in the overall structure of the protein surely attest to better functionalities.

Based on molecular docking features of quercetin (a powerful antioxidant flavonoid) Ji *et*. *al*. [21] proposed that quercetin binds the Nrf2-binding site of Keap, which prevents Keap1 association with Nrf2. Since association of Nrf2 with Keap1 keeps Nrf2 inactive [19], hence, quercetin facilitates activation of Nrf2 and Nrf2-regulated genes such as HO-1. Our molecular docking studies on Keao1-Nrf2 showed significant differences between 3HF and 7HF (Table 1), which may imply a similar interaction between 7HF and Keap1. The consequence of such an interaction is an augmented Nrf2 activation by 7HF and consequently Nrf2-mediated upregulation of HO-1 (Fig 2). Further studies are needed to verify this scenario.

**Fig 6:**
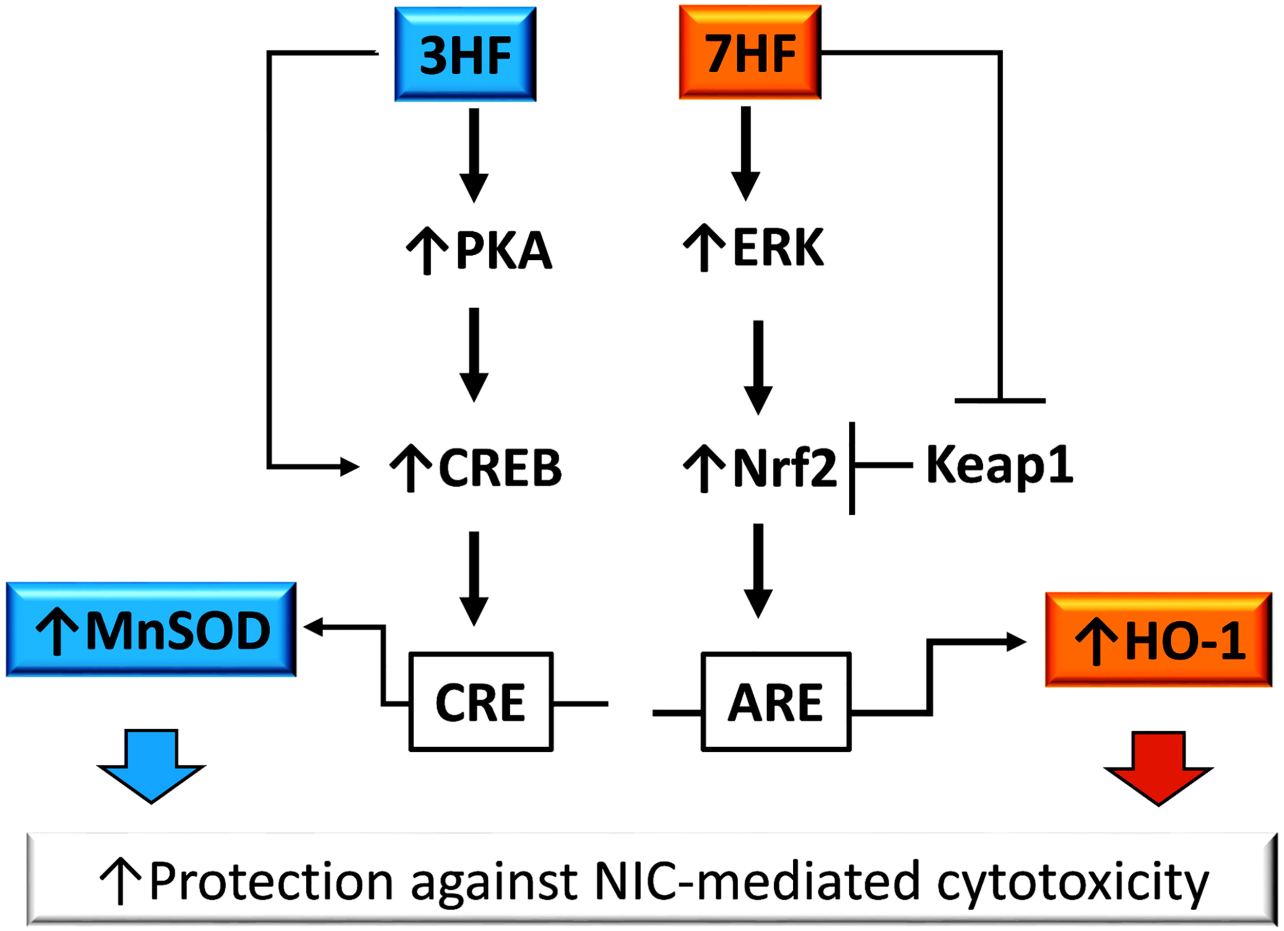
An intriguing difference in the pathways of the protective response. Flavonol 3-hydroxyflavone (3HF) activates the antioxidant MnSOD gene via the PKA/CREB pathway, which is potentially augmented by high affinity binding of 3HF to CREB. In contrast, 7-hydroxyflavone (7HF) upregulates the HO-1 gene via the ERK/Nrf2 axis, which may be facilitated by 7HF binding to the Nrf2-binding domain of Keap1 that releases Nrf2 from Keap1 resulting in activation of Nrf2.

Our docking studies on CREB protein 1DH3 (Table 1, Fig 4) identified that 3HF binds in the region made of TYR336- LYS330- LYS333- LEU332- LEU329, which shows higher binding affinity than 7HF (where binding site is made of LYS330- LYS333- LEU332- LEU329 amino acids). Interestingly, the binding pocket is different than ATP binding site [53] suggesting no interference between the two. These implies that the high affinity binding of 3HF to CREB protein may facilitate its phosphorylation allosterically, which could interpret the observed CRE activation by 3HF (Fig 2C) and the consequent MnSOD activation by CREB (Fig 2A). Fig 6 summarizes the study by highlighting the difference in the mechanism underlying the protective effects of two monohydroxyflavone isomers against nicotine induced stress. Future studies using competitive inhibitors of ATP in the presence of flavones are needed to attest our interpretations.

The uniqueness of this study lies in the fundamentals of understanding of the mechanism of cellular protective functions of a single hydroxyl group in flavone, and the importance of its position in the structure. The wide applications of this promising approach would likely to open an avenue for the screening and design of the most suitable flavonoid derivatives among other structural variants of this new generation of rapidly emerging potential therapeutic drugs.

## Supporting information

S1 Fig RMSF of free and bound proteins over residues. Root mean squared fluctuation over residues numbers for unliganded and complexed (with 3HF and 7HF) proteins: A. cAMP responsive element-binding protein 1DH3, and B. Keap1-Nrf2 conjugate protein 2DYH.
(TIF)

S2 Fig RMSF of bound proteins over atoms. Root mean squared fluctuation over atom numbers for complexed proteins: A. cAMP responsive element-binding protein 1DH3, and B. Keap1-Nrf2 conjugate protein 2DYH, with 3HF and 7HF.
(TIF)
