# Supplementary figures and images for: Differential roles of 3-Hydroxyflavone and 7-Hydroxyflavone against nicotine-induced oxidative stress in rat renal proximal tubule cells
Source: PLoS One. 2017 Jun 22;12(6):e0179777. doi: 10.1371/journal.pone.0179777 (PMC5480997; doi:10.1371/journal.pone.0179777)

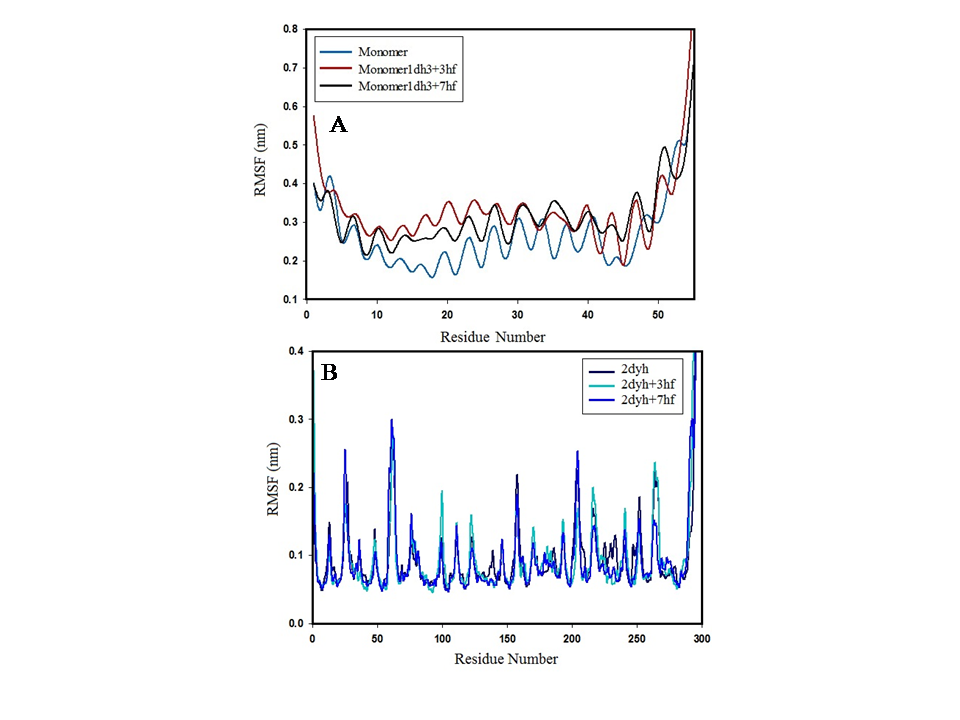

Supplement: S1 Fig — Root mean squared fluctuation over residues numbers for unliganded and complexed (with 3HF and 7HF) proteins: A. cAMP responsive element-binding protein 1DH3, and B. Keap1-Nrf2 conjugate protein 2DYH. (TIF) [file pone.0179777.s001.tif]

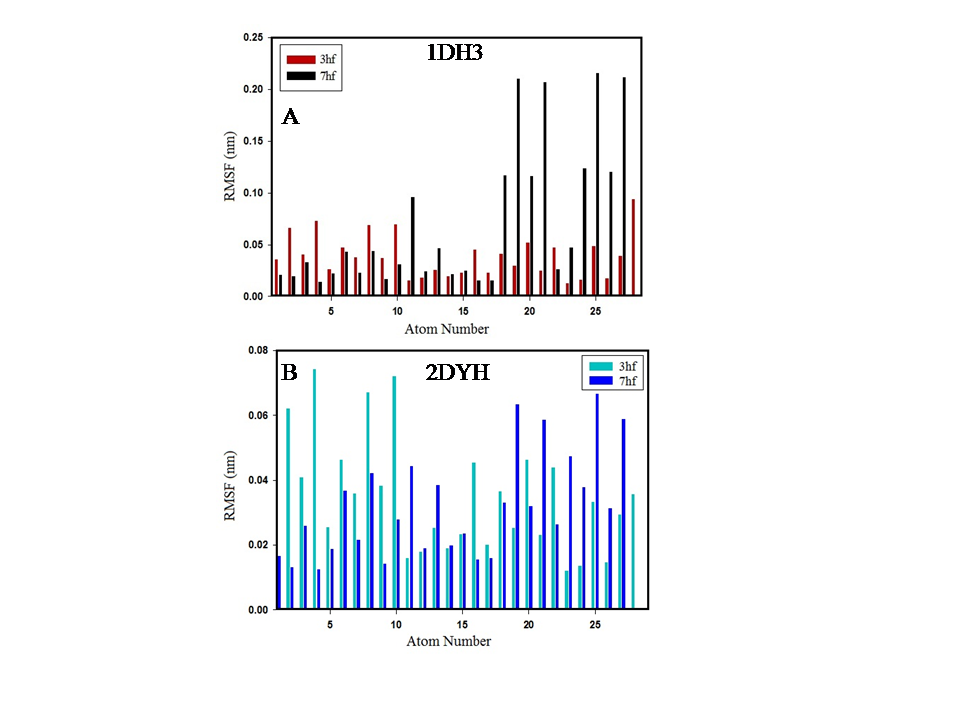

Supplement: S2 Fig — Root mean squared fluctuation over atom numbers for complexed proteins: A. cAMP responsive element-binding protein 1DH3, and B. Keap1-Nrf2 conjugate protein 2DYH, with 3HF and 7HF. (TIF) [file pone.0179777.s002.tif]
